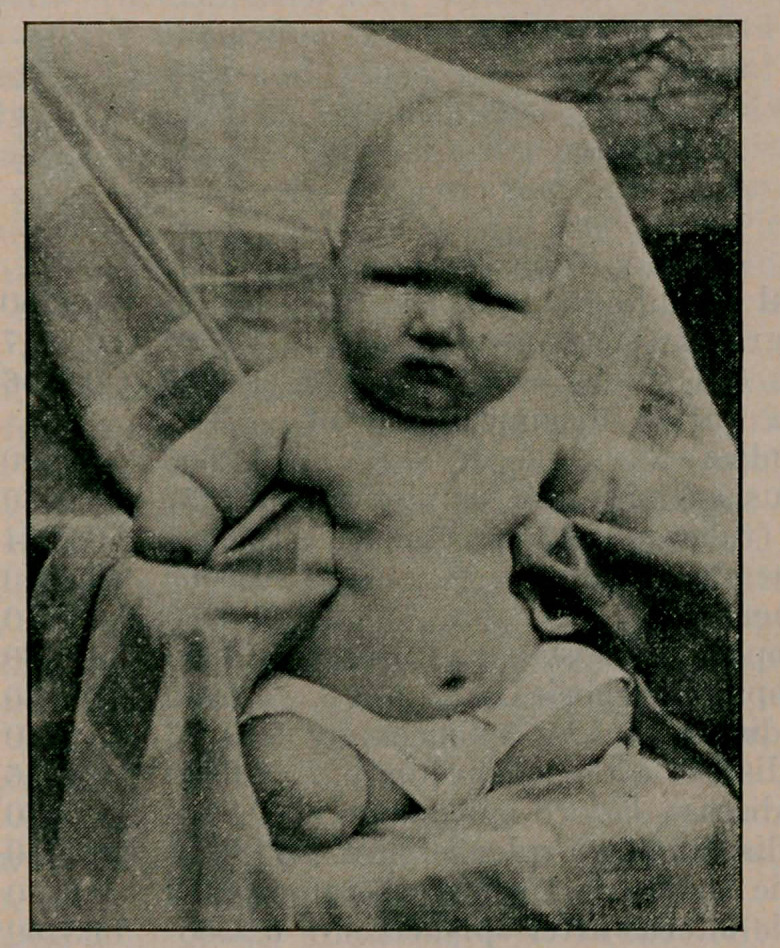# Baby without Hands or Feet

**Published:** 1915-08

**Authors:** 


**Baby Without Hands or Feet.** W. H. Malone of Tallapoosa, Ga., Med. World July, reports the following case of a male child, born at term, weight 8 pounds, healthy at 6 months. The mother was a white, unmarried primipara, aged 19.

## Figures and Tables

**Figure f1:**